# Regulation of Secondary Metabolism by the Velvet Complex Is Temperature-Responsive in *Aspergillus*

**DOI:** 10.1534/g3.116.033084

**Published:** 2016-09-30

**Authors:** Abigail L. Lind, Timothy D. Smith, Timothy Saterlee, Ana M. Calvo, Antonis Rokas

**Affiliations:** * Department of Biomedical Informatics, Vanderbilt University Medical Center, Nashville, Tennessee 37203; † Department of Biological Sciences, Northern Illinois University, DeKalb, Illinois 60115; ‡ Department of Biological Sciences, Vanderbilt University, Nashville, Tennessee 37232

**Keywords:** *Aspergillus fumigatus*, gene regulation, temperature-dependent gene expression, gene cluster

## Abstract

Sensing and responding to environmental cues is critical to the lifestyle of filamentous fungi. How environmental variation influences fungi to produce a wide diversity of ecologically important secondary metabolites (SMs) is not well understood. To address this question, we first examined changes in global gene expression of the opportunistic human pathogen, *Aspergillus fumigatus*, after exposure to different temperature conditions. We found that 11 of the 37 SM gene clusters in *A. fumigatus* were expressed at higher levels at 30° than at 37°. We next investigated the role of the light-responsive Velvet complex in environment-dependent gene expression by examining temperature-dependent transcription profiles in the absence of two key members of the Velvet protein complex, VeA and LaeA. We found that the 11 temperature-regulated SM gene clusters required VeA at 37° and LaeA at both 30 and 37° for wild-type levels of expression. Interestingly, four SM gene clusters were regulated by VeA at 37° but not at 30°, and two additional ones were regulated by VeA at both temperatures but were substantially less so at 30°, indicating that the role of VeA and, more generally of the Velvet complex, in the regulation of certain SM gene clusters is temperature-dependent. Our findings support the hypothesis that fungal secondary metabolism is regulated by an intertwined network of transcriptional regulators responsive to multiple environmental factors.

Filamentous fungi produce a diverse array of small molecules collectively known as secondary metabolites (SMs). Much research on SMs has focused on their double-edged impact on humans (Keller *et al.* 2005); many are valued as pharmaceuticals, such as the antibiotic penicillin and the cholesterol-lowering drug lovastatin (Kennedy *et al.* 1999; Paláez 2004), whereas others are potent toxins, such as the acutely carcinogenic aflatoxin (Bennett and Klich 2003). In the fungal natural environment, SMs have a variety of functions: they can operate as signaling molecules (Yim *et al.* 2007; Rodríguez-Urra *et al.* 2012), as virulence factors to aid pathogenic lifestyles (Proctor *et al.* 1995; Stanzani *et al.* 2005; Coméra *et al.* 2007), as microbial inhibitors to carve out a competitive advantage in environments crowded with other microbes (Losada *et al.* 2009; König *et al.* 2013), or as a defense against fungivorous predators (Rohlfs *et al.* 2007; Calvo and Cary 2015). SM production is closely linked with environmental signals (Brakhage *et al.* 2009; Keller 2015); for example, the SM aflatoxin is not produced by *Aspergillus parasiticus* at 37°, the organism’s optimal temperature for growth, but is produced at 28° (Feng and Leonard 1998). Furthermore, the effects of specific environmental conditions on SM production can be varied; for example, sterigmatocystin is produced in much higher quantities at 37° than at 28° in *A. nidulans*, a pattern of expression that is the reverse of its close chemical relative aflatoxin in *A. parasiticus* (Feng and Leonard 1998).

The expression of genes involved in the synthesis and secretion of SMs is governed by a hierarchical network of master regulators that respond to multiple environmental cues (Brakhage 2013). One such environmentally responsive complex of master regulators is the Velvet protein complex, whose constituent proteins are broadly conserved regulators of fungal development and secondary metabolism (Bayram and Braus 2012; Calvo *et al.* 2016). In the absence of light in *A. nidulans*, two Velvet complex members, VeA and VelB, enter the nucleus, where VeA interacts with the chromatin-modifying protein LaeA (Bayram *et al.* 2008). The resulting heterotrimeric protein complex modulates expression of SM gene clusters and developmental processes in many fungi (Wiemann *et al.* 2010; Hoff *et al.* 2010; Chettri *et al.* 2012; Lind *et al.* 2015), including the opportunistic human pathogen *A. fumigatus* (Perrin *et al.* 2007; Dhingra *et al.* 2012, 2013).

While most master regulators of secondary metabolism are known in the context of the individual environmental cues that activate them, it is likely that these regulators combinatorically control SM production to fine-tune the metabolic profile of a fungus to changing environments. The possibility of combinatorial regulation is supported by recent studies showing that multiple environmental cues can regulate production of the SM terrain in *A. terreus* (Gressler *et al.* 2015), that both the light-responsive regulator VeA and the nitrogen regulator AreA are required for wild-type (WT) levels of SM-producing gene transcription in *Fusarium oxysporum* (López-Berges *et al.* 2014), and that glucose concentration can impact SM production in *A. nidulans* through changes in the subcellular localization of VeA (Atoui *et al.* 2010).

The fungal genus *Aspergillus* is an excellent system to examine the influence of environmental variation in SM regulation, as the mechanisms for SM production have been widely studied in this group of organisms. The SM gene clusters (Inglis *et al.* 2013) and SM production profiles (Chiang *et al.* 2008; Frisvad *et al.* 2009) of several species are described in depth, and several master SM regulators are well characterized (Brakhage 2013). Furthermore, although variation of SM production in response to environmental cues, including temperature (O’Brian *et al.* 2007; Yu *et al.* 2011), pH (Tilburn *et al.* 1995; Bignell *et al.* 2005), light (Bayram *et al.* 2008), and hypoxia (Blatzer *et al.* 2011; Barker *et al.* 2012) has been observed, it has not been systematically characterized or mechanistically understood. For this study, we chose *A. fumigatus*, the most common cause of a suite of diseases known collectively as aspergillosis (Latge 1999). *A. fumigatus* produces a diverse array of SMs, including the immune-suppressing SM gliotoxin, which is thought to promote its virulence (Scharf *et al.* 2012). Additionally, *A. fumigatus* is highly thermotolerant; it can grow at 55° and can survive at temperatures up to 75° (Beffa *et al.* 1998; Ryckeboer *et al.* 2003; Abad *et al.* 2010). It is unknown whether changes in temperature affect global patterns of gene expression in the secondary metabolic pathways of this opportunistic pathogen.

To test whether variation in environmental cues other than the known light response can influence Velvet complex–based SM regulation in *A. fumigatus*, we examined global gene expression using RNA sequencing (RNA-seq) in response to different temperatures in WT, Δ*veA*, and Δ*laeA* backgrounds. We found that change in temperature had a marked impact on the expression of SM genes, and that VeA regulates the genes required for producing at least four SMs at 37 but not at 30°, suggesting that the Velvet complex is involved in both temperature- and light-based regulation of secondary metabolism in *Aspergillus*.

## Materials and Methods

### Strains and culture conditions

*A. fumigatus* WT CEA10, Δ*veA* TDS1.15 (*pyrG1* Δ*veA* ::*pyrG^A. fum^*) (Dhingra *et al.* 2012), and TSD62.1 (*pyrG1* Δ*veA* ::*pyrG^A. fum^*) (Dhingra *et al.* 2013) were used in this study. Strains were stored as 30% glycerol stocks at −80°. Conidia of *A. fumigatus* WT, Δ*veA*, and Δ*laeA* strains were inoculated in 25 ml Czapek–Dox medium (10^7^/ml) and grown as stationary cultures for 72 hr at either 30 or 37° in the dark.

### RNA isolation

Mycelial mats were collected and immediately frozen in liquid nitrogen. Samples were then lyophilized and ground. Total RNA was extracted using Direct-zol RNA MiniPrep Kit from ZYMO, following the manufacturer’s instructions. RNA was resuspended in autoclaved double-distilled H_2_O. Samples were stored at −80°. Expected *veA* and *laeA* expression patterns in the WT and corresponding deletion mutants were verified by quantitative RT-PCR (qRT-PCR; Supplemental Material, Figure S2).

### RNA-seq

RNA-seq libraries were constructed and sequenced at the Vanderbilt Technologies for Advanced Genomics Core Facility at Vanderbilt University, using the Illumina Tru-seq RNA sample prep kit, as previously described (Dhingra *et al.* 2012; Lind *et al.* 2015). Briefly, total RNA quality was assessed via Bioanalyzer (Agilent Technologies). Upon passing quality control, poly-A RNA was purified from total RNA and second-strand complementary DNA (cDNA) was synthesized from messenger RNA. cDNA ends were then blunt repaired and 3′ ends were adenylated. Barcoded adapters were ligated to the adenylated ends and the libraries were PCR-enriched, quantified, pooled, and sequenced on an Illumina HiSequation 2500 sequencer. Two biological replicates were generated for each strain sequenced.

### Gene expression analysis

Raw RNA-seq reads were trimmed of low-quality reads and adapter sequences using Trimmomatic with the suggested parameters for single-end read trimming (Bolger *et al.* 2014). After read trimming, all samples contained between 9.5 and 14.1 million reads, with the average sample containing 12 million reads. Trimmed reads were aligned to the *A. fumigatus* Af293 version s03_m04_r11 genome from the *Aspergillus* Genome Database (Arnaud *et al.* 2010, 2012). Read alignment was performed with Tophat2, using the reference gene annotation to guide alignment and without attempting to detect novel transcripts (parameter: –no-novel-juncs) (Kim *et al.* 2013). Reads aligning to each gene were counted using HTSeq-count, with the union mode (Anders *et al.* 2014). Differential expression was determined using the DESeq2 *R* package (Love *et al.* 2014). Genes were considered differentially expressed if their Benjamini–Hochberg adjusted p-value was < 0.1 and their log_2_ fold-change was > 1 or < −1.

### Functional enrichment analysis

Functional category enrichment was determined for overexpressed and underexpressed genes in all conditions tested, using the Cytoscape plugin BiNGO (Shannon *et al.* 2003; Maere *et al.* 2005). To allow for a high-level view of the types of differentially expressed gene sets, the *Aspergillus* GOSlim v1.2 term subset was used (The Gene Ontology Consortium 2014). The Benjamini–Hochberg multiple testing correction was applied and functional categories were considered significantly enriched if the adjusted p-value was < 0.05.

### Gene cluster expression

*A. fumigatus* secondary metabolic gene clusters were taken from a combination of computationally predicted and experimentally characterized gene clusters (Inglis *et al.* 2013; Lind *et al.* 2015). A list of all SM gene clusters used in this study is available in Table 1. SM gene clusters were designated as differentially expressed if half or more of the genes in the cluster were differentially expressed.Gene clusters where half or more genes were significantly differentially expressed (adjusted p-value < 0.1) but with a |log_2_ fold change| less than 1 were considered weakly differentially expressed. Clusters containing a mix of overexpressed and underexpressed genes were considered to have mixed expression.

**Table 1:** All secondary metabolic clusters in *A. fumigatus*

Cluster Number	Cluster Name/Product	Cluster Genes	Reference
Cluster 1	Not known	Afu1g00980, Afu1g00990, Afu1g01000, Afu1g01010	Inglis *et al.* (2013)
Cluster 2	Not known	Afu1g10270, Afu1g10280, Afu1g10295, Afu1g10310, Afu1g10320, Afu1g10330, Afu1g10340, Afu1g10350, Afu1g10355, Afu1g10360, Afu1g10370, Afu1g10380	Inglis *et al.* (2013)
Cluster 3	Not known	Afu1g17200, Afu1g17210, Afu1g17220, Afu1g17230, Afu1g17240	Inglis *et al.* (2013)
Cluster 4	Not known	Afu1g17710, Afu1g17720, Afu1g17723, Afu1g17725, Afu1g17730, Afu1g17740	Inglis *et al.* (2013)
Cluster 5	Not known	Afu2g01280, Afu2g01290, Afu2g01300, Afu2g01310, Afu2g01320, Afu2g01330	Inglis *et al.* (2013)
Cluster 6	Not known	Afu2g05740, Afu2g05750, Afu2g05760, Afu2g05770, Afu2g05780, Afu2g05790, Afu2g05800, Afu2g05810, Afu2g05820, Afu2g05830	Inglis *et al.* (2013)
Cluster 7	1,8-Dihydroxynaphthalene (DHN) melanin	Afu2g17530, Afu2g17540, Afu2g17550, Afu2g17560, Afu2g17580, Afu2g17600	Tsai *et al.* (1999)
Cluster 8	Fumigaclavine	Afu2g17960, Afu2g17970, Afu2g17980, Afu2g17990, Afu2g18000, Afu2g18010, Afu2g18020, Afu2g18030, Afu2g18040, Afu2g18050, Afu2g18060	Robinson and Panaccione (2012)
Cluster 9	Not known	Afu3g01400, Afu3g01410, Afu3g01420, Afu3g01430, Afu3g01440, Afu3g01450, Afu3g01460, Afu3g01470, Afu3g01480	Inglis *et al.* (2013)
Cluster 10	Not known	Afu3g02520, Afu3g02530, Afu3g02540, Afu3g02550, Afu3g02560, Afu3g02570, Afu3g02580, Afu3g02585, Afu3g02590, Afu3g02600, Afu3g02610, Afu3g02620, Afu3g02630, Afu3g02640, Afu3g02650	Inglis *et al.* (2013)
Cluster 11	Not known	Afu3g02670, Afu3g02680, Afu3g02685, Afu3g02690, Afu3g02700, Afu3g02710, Afu3g02720	Inglis *et al.* (2013)
Cluster 12	Not known	Afu3g03300, Afu3g03310, Afu3g03315, Afu3g03320, Afu3g03330, Afu3g03340, Afu3g03350, Afu3g03370, Afu3g03380, Afu3g03390, Afu3g03400, Afu3g03410, Afu3g03420, Afu3g03430, Afu3g03440, Afu3g03445, Afu3g03450, Afu3g03460	Inglis *et al.* (2013)
Cluster 13	Hexadehydroastechrome (HAS) cluster	Afu3g12890, Afu3g12900, Afu3g12910, Afu3g12920, Afu3g12930, Afu3g12940, Afu3g12950, Afu3g12960	Yin *et al.* (2013)
Cluster 14	Not known	Afu3g13670, Afu3g13680, Afu3g13690, Afu3g13700, Afu3g13710, Afu3g13720, Afu3g13730, Afu3g13740, Afu3g13750	Inglis *et al.* (2013)
Cluster 15	Not known	Afu3g14690, Afu3g14700, Afu3g14710, Afu3g14720, Afu3g14730	Inglis *et al.* (2013)
Cluster 16	Not known	Afu3g15240, Afu3g15250, Afu3g15260, Afu3g15270, Afu3g15280, Afu3g15290	Inglis *et al.* (2013)
Cluster 17	Endocrocin	Afu4g00210, Afu4g00220, Afu4g00225, Afu4g00230	Lim *et al.* (2012)
Cluster 18	Not known	Afu4g11170, Afu4g11180, Afu4g11190, Afu4g11200, Afu4g11210, Afu4g11220, Afu4g11230, Afu4g11240, Afu4g11250, Afu4g11260, Afu4g11270, Afu4g11280, Afu4g11290, Afu4g11300	Inglis *et al.* (2013)
Cluster 19	Not known	Afu4g11980, Afu4g11990, Afu4g12000, Afu4g12010, Afu4g12020, Afu4g12030, Afu4g12040, Afu4g12050, Afu4g12060, Afu4g12070	Inglis *et al.* (2013)
Cluster 20	Trypacidin	Afu4g14460, Afu4g14480, Afu4g14470, Afu4g14490, Afu4g14500, Afu4g14510, Afu4g14520, Afu4g14530, Afu4g14540, Afu4g14550, Afu4g14560, Afu4g14570, Afu4g14580	Throckmorton *et al.* (2015); Mattern *et al.* (2015)
Cluster 21	Not known	Afu5g00100, Afu5g00110, Afu5g00120, Afu5g00130, Afu5g00135	Inglis *et al.* (2013)
Cluster 22	Not known	Afu5g10040, Afu5g10050, Afu5g10060, Afu5g10070, Afu5g10080, Afu5g10090, Afu5g10100, Afu5g10110, Afu5g10120, Afu5g10130	Inglis *et al.* (2013)
Cluster 23	Not known	Afu5g12730, Afu5g12740, Afu5g12750, Afu5g12760, Afu5g12770	Inglis *et al.* (2013)
Cluster 24	Fumipyrrole	Afu6g03430, Afu6g03440, Afu6g03450, Afu6g03460, Afu6g03470, Afu6g03480, Afu6g03490	Macheleidt *et al.* (2015)
Cluster 25	Not known	Afu6g08550, Afu6g08560	Inglis *et al.* (2013)
Cluster 26	Gliotoxin	Afu6g09630, Afu6g09640, Afu6g09650, Afu6g09660, Afu6g09670, Afu6g09680, Afu6g09690, Afu6g09700, Afu6g09710, Afu6g09720, Afu6g09730, Afu6g09740	Gardiner and Howlett (2005)
Cluster 27	Fumiquinazoline	Afu6g12040, Afu6g12050, Afu6g12060, Afu6g12070, Afu6g12080	Ames *et al.* (2010)
Cluster 28	Not known	Afu6g13920, Afu6g13930, Afu6g13940, Afu6g13945, Afu6g13950, Afu6g13970, Afu6g13980, Afu6g13990, Afu6g14000	Inglis *et al.* (2013)
Cluster 29	Neosartoricin/fumicycline	Afu7g00120, Afu7g00130, Afu7g00150, Afu7g00160, Afu7g00170, Afu7g00180, Afu7g00190	König *et al.* (2013)
Cluster 30	Not known	Afu7g00260, Afu7g00270	Inglis *et al.* (2013)
Cluster 31	Not known	Afu7g01180, Afu7g01190, Afu7g01200, Afu7g01210, Afu7g01220, Afu7g01230, Afu7g01240, Afu7g01250, Afu7g01260, Afu7g01270	Inglis *et al.* (2013)
Cluster 32	Fumitremorgin	Afu8g00170, Afu8g00190, Afu8g00200, Afu8g00210, Afu8g00220, Afu8g00230, Afu8g00240, Afu8g00250, Afu8g00260	Maiya *et al.* (2006)
Cluster 33	Fumagillin	Afu8g00370, Afu8g00380, Afu8g00390, Afu8g00400, Afu8g00410, Afu8g00420, Afu8g00430, Afu8g00440, Afu8g00460, Afu8g00470, Afu8g00480, Afu8g00490, Afu8g00500, Afu8g00510, Afu8g00520	Lin *et al.* (2013)
Cluster 34	Pseurotin	Afu8g00530, Afu8g00540, Afu8g00550, Afu8g00560, Afu8g00570, Afu8g00580	Wiemann *et al.* (2013)
Cluster 35	Not known	Afu8g00590, Afu8g00595, Afu8g00600, Afu8g00610, Afu8g00620, Afu8g00630, Afu8g00640	Inglis *et al.* (2013)
Cluster 36	Not known	Afu8g01630, Afu8g01640	Inglis *et al.* (2013)
Cluster 37	Not known	Afu8g02350, Afu8g02360, Afu8g02380, Afu8g02390, Afu8g02400, Afu8g02410, Afu8g02420, Afu8g02430	Inglis *et al.* (2013)

### Temperature-shift experiments

Conidia from WT strains were inoculated in Czapek–Dox (10^7^ spores/ml) and grown as liquid shake cultures in the dark at 30°. After 24 hr of growth, equal biomass (1 g) was transferred to new flasks, which were then cultured at 30 or 37°. Mycelia were harvested and RNA extracted as previously described, at 24 and 72 hr time points, with three biological replicates. This temperature-shift experiment was also performed with a starting culture temperature of 37°.

For expression analysis, 5 μg of total RNA was treated with RQ1 RNase-Free DNase (Promega, Madison, WI). cDNA was synthesized with Moloney murine leukemia virus reverse transcriptase (Promega). qRT-PCR was performed with the Applied Biosystems 7000 Real-Time PCR System, using SYBR green dye for fluorescence detection. To determine expression values, cDNA was normalized to 18S ribosomal gene expression. Expression of two backbone biosynthetic genes, gliP and psoA of the gliotoxin and pseurotin gene clusters, was assayed using the primers in Table S1.

### Data availability

All RNA-seq data files are available from the NCBI’s Short Read Archive database (accession number: SRP080951).

## Results

### Temperature shift changes the expression of 10% of all genes and of more than half of the genes in SM gene clusters

**Figure 1:**
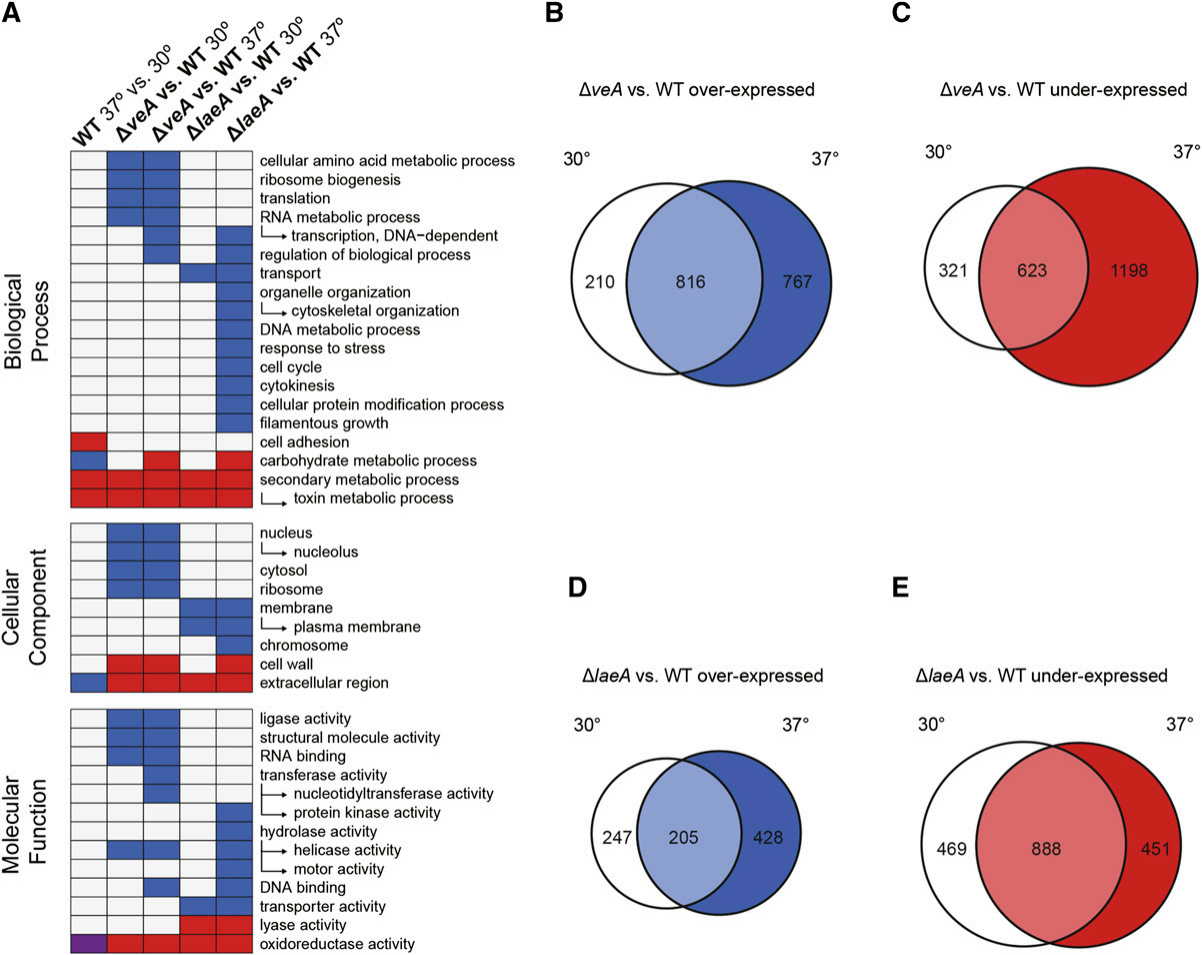
Comparison of enriched functional categories and overlapping differential gene expression. (A) Enriched functional categories for genes differentially expressed under variable temperature conditions. Red boxes indicate GOSlim terms enriched in underexpressed genes, blue boxes indicate categories enriched in overexpressed genes, and purple boxes indicate categories enriched in both overexpressed and underexpressed genes. (B and C) Overlap between genes differentially expressed in Δ*veA vs.* WT at 30 and 37°. (D and E) Overlap between genes differentially expressed in Δ*laeA vs.* WT at 30 and 37°.

To investigate the effect of temperature on gene expression, we compared the transcriptomes of *A. fumigatus* WT grown at 37° Compared with WT grown at 30°. This comparison identified 1101 differentially expressed genes (log_2_ fold-change > 1, adjusted p-value < 0.1), which corresponds to > 10% of the *A. fumigatus* transcriptome. Of these genes, 402 were expressed at a higher degree (overexpressed) and 699 genes were expressed at a lower degree (underexpressed) at 37° than at 30° (File S1). Genes overexpressed at 37° were enriched (adjusted p-value < 0.05) for the functional categories carbohydrate metabolic process and extracellular region; genes underexpressed at 37° were enriched for the categories cell adhesion, secondary metabolic process, toxin metabolic process, and oxidoreductase activity (Figure 1A).

As functional category enrichment analysis indicated that genes involved in secondary metabolism were expressed at lower levels in WT at 37° than at 30°, we next investigated the impact of temperature on expression of each of the 37 previously identified secondary metabolic gene clusters (Inglis *et al.* 2013; Lind *et al.* 2015). We found that half or more of the genes in 13 gene clusters were expressed at lower levels at 37° than at 30°, including the clusters encoding the conidial melanin pigment, fumigaclavine, endocrocin, trypacidin, fumipyrrole, gliotoxin, fumiquinazoline, fumitremorgin, fumagillin, pseurotin, and three gene clusters that do not encode known products (cluster 15, cluster 30, and cluster 35) (Figure 3 and File S1). As previous analysis has shown that endocrocin is not produced at temperatures above 35°, these results indicate that this is attributable to changes in gene expression (Berthier *et al.* 2013).Three other gene clusters that do not encode known products, namely cluster 21, cluster 25, and cluster 36, were overexpressed at 37° (Figure 3 and File S1). Additionally, half or more genes in six gene clusters (cluster 5, cluster 6, cluster 18, cluster 23, cluster 28, and cluster 31) were differentially expressed but contained a mixture of both overexpressed and underexpressed genes; none of these gene clusters encode known products.

The effect of temperature on SM production on two of these gene clusters, gliotoxin and pseurotin, were further tested using temperature-shift experiments. Cultures were grown at 30° and then shifted to either 37 or 30° and harvested after 24 and 72 hr of growth. The shift experiment was also performed by growing the starting culture at 37° and then shifting to either 30 or 37°. Recapitulating our RNA-seq based results, the backbone synthesis gene from the gliotoxin gene cluster, gliP, was more highly expressed at 30 than 37° at 24 and 72 hr in both temperature up- and down-shift experiments (Figure S3A). The backbone synthesis gene from the pseurotin gene cluster, psoA, was more highly expressed at 37 than 30° at the 24 hr time point during the temperature up-shift experiment; however, at 72 hr, the gene was more highly expressed at 37° (Figure S3B). Further, psoA was more highly expressed at 30 than 37° at all time points for temperature down-shift experiments.

### VeA regulates a much large number of genes at 37° than at 30°

To investigate how temperature influences VeA’s role in controlling gene expression, we compared the transcriptomes of a Δ*veA* strain with WT grown at either 37 or 30°. In agreement with previous studies (Dhingra *et al.* 2013; Lind *et al.* 2015), we found a very large number (3404) of differentially expressed genes in Δ*veA* at 37°, with 1821 overexpressed genes and 1583 underexpressed genes (File S1). Far fewer genes were differentially expressed in the Δ*veA* strain at 30°. Specifically, 1986 genes were differentially expressed in Δ*veA*, with 1026 genes overexpressed and 960 genes underexpressed (File S1). A comparison of the 3404 differentially expressed genes at 37° with the 1986 differentially expressed genes at 30° revealed that a subset of 1468 genes were differentially expressed in Δ*veA* at both temperatures, suggesting that their regulation by VeA is temperature independent (Figure 1B). However, while 518 genes were differentially expressed solely at 30°, almost four times as many genes (1935) were differentially expressed solely at 37°; these results indicate that the regulatory impact of VeA is much greater at 37° than at 30°.

To determine the functions of differentially expressed genes in Δ*veA* *vs.* WT at 30 and 37°, we performed functional category enrichment analyses. Overexpressed genes in Δ*veA* were enriched for functional categories relating to transcription and translation activity at both 30 and 37°, while the categories DNA-dependent transcription, transferase activity, nucleotidyltransferase activity, regulation of biological process, and DNA binding were only enriched at 37° (Figure 1A). Further, the number of overexpressed genes in each category was higher for all significantly enriched categories at 37°, with the exceptions of lyase activity, cytoskeletal organization, and motor activity, which were unchanged (File S2).

Genes underexpressed in Δ*veA* were enriched for functional categories related to secondary metabolism, including secondary metabolic process and toxin metabolic process. The only significantly enriched category for genes underexpressed in Δ*veA* at 37° that was not enriched for genes underexpressed at 30° was carbohydrate metabolic process (Figure 1A). The number of underexpressed genes annotated to each functional category was higher at 37°, with the exception of ribosome, cytoskeletal organization, and cell adhesion, which remained unchanged (File S2). These enrichment analyses indicate that though many more genes are differentially expressed in Δ*veA* at 37°, VeA is regulating similar categories of genes at both temperatures.

### LaeA regulates similar numbers and types of genes at 30° and 37°

To investigate how temperature influences LaeA’s role in gene regulation, we compared the transcriptomes of a Δ*laeA* strain with WT grown at either 37 or 30°. While Δ*veA* strains showed temperature-dependent differences in the number of differentially expressed genes, Δ*laeA* strains showed similar numbers of differentially expressed genes at both 30 and 37°. In total, 1971 genes were differentially expressed in Δ*laeA* strains compared with WT at 37° (632 overexpressed and 1339 underexpressed), while 1809 genes were differentially expressed at 30° (452 overexpressed and 1357 underexpressed) (File S1). There was moderate overlap of the sets of differentially expressed genes at the two temperatures; 1109 genes were differentially expressed in Δ*laeA* at both temperatures, while 770 and 862 genes were only differentially expressed at 30 and 37°, respectively (Figure 1C).

To identify the functions of genes differentially expressed in the Δ*laeA* strain compared with WT at 37 and 30°, we performed functional category enrichment analyses. Genes overexpressed at both 37 and 30° in Δ*laeA* were enriched for the categories transport, transporter activity, membrane, and plasma membrane. However, genes overexpressed at 37° were enriched for an additional 14 functional categories related to cell division, filamentous growth, and DNA metabolism that were not enriched in genes overexpressed at 30° (Figure 1A). Underexpressed genes at both 30 and 37° were enriched for categories relating to secondary metabolism, in agreement with LaeA’s well-documented role as a master regulator of secondary metabolism (Bok *et al.* 2006; Bayram and Braus 2012). Two functional categories, carbohydrate metabolism and cell wall, were enriched for underexpressed genes at 30 but not 37°.

### VeA and LaeA have greater regulatory overlap at 37° than at 30°

**Figure 2:**
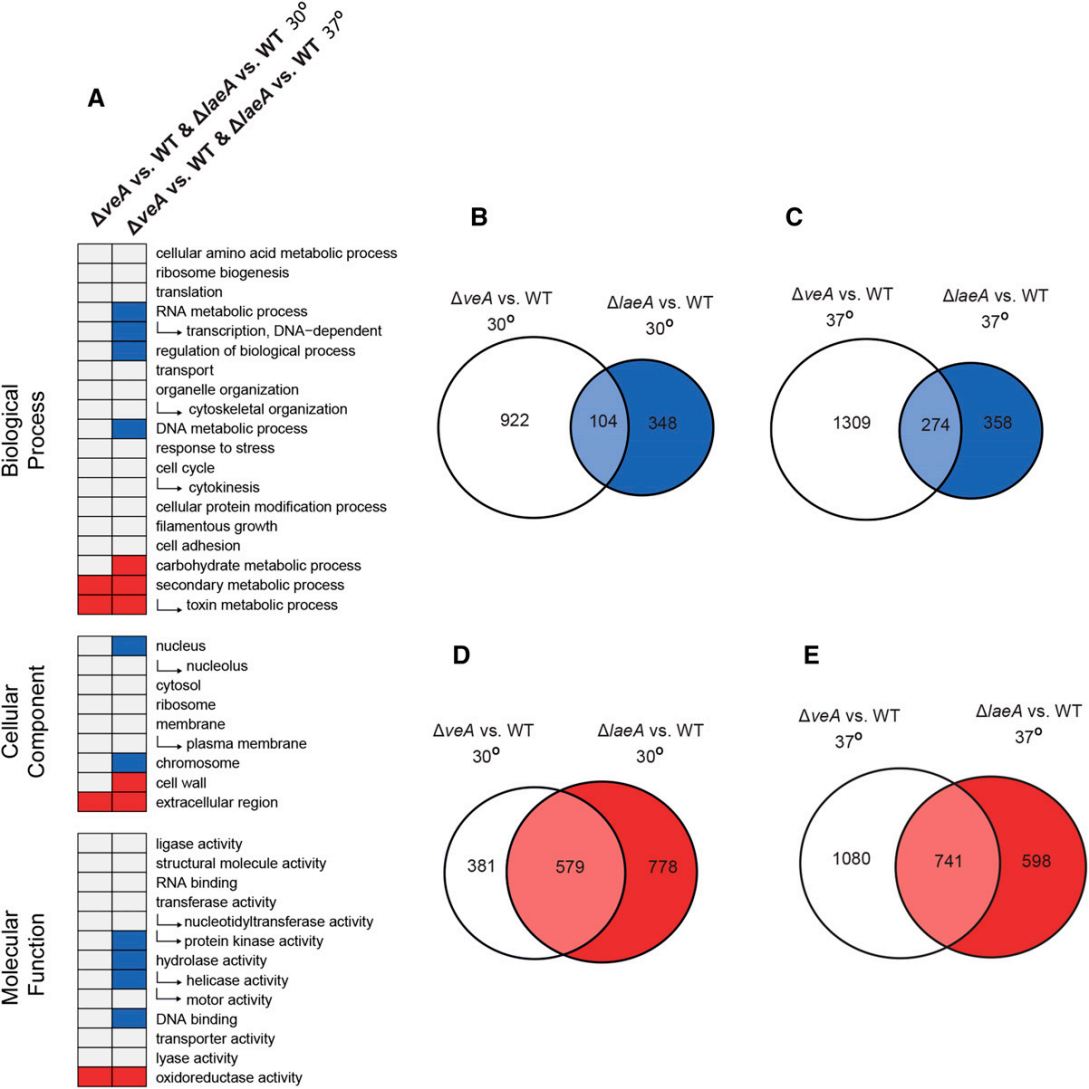
Comparison of enriched functional categories and differential gene expression in Δ*veA* and Δ*laeA* strains at 30° and 37°. (A) Enriched functional categories for genes differentially expressed in both Δ*veA* vs. WT and in Δ*laeA* vs. WT at either 30° or 37°. Red boxes indicate GOSlim terms enriched in under-expressed genes and blue boxes indicate categories enriched in over-expressed genes. No categories were enriched for both over- and under-expressed genes. (B) Overlap between genes over-expressed in Δ*veA* vs. WT and Δ*laeA* vs. WT at 30°. (C) Overlap between genes over-expressed in Δ*veA* vs. WT and Δ*laeA* vs. WT at 37°. (D) Overlap between genes under-expressed in Δ*veA* vs. WT and Δ*laeA* vs. WT at 30°. (E) Overlap between genes under-expressed in Δ*veA* vs. WT and Δ*laeA* vs. WT at 37°.

As VeA and LaeA are both members of the Velvet complex and are known to interact, it is very likely that they exhibit substantial overlap in the genes they regulate (Calvo 2008). To examine the effect of temperature on this regulatory overlap, we determined the intersection of genes differentially expressed in Δ*veA* *vs.* WT and Δ*laeA vs.* WT at 30 and 37°. In total, 741 genes were underexpressed in both Δ*veA* and Δ*laeA* at 37° (this number corresponds to 41% of all underexpressed genes in Δ*veA* and 55% of all underexpressed genes in Δ*laeA*) and 579 genes were underexpressed in both Δ*veA* and Δ*laeA* at 30° (41% of all underexpressed genes in Δ*veA* and 55% of all underexpressed genes in Δ*laeA*) (Figure 2, B and C). The 741 genes underexpressed at 37° were significantly enriched for the functional categories secondary metabolic process, oxidoreductase activity, extracellular region, toxin metabolic process, cell wall, and carbohydrate metabolic process (Figure 2A and File S3). The 579 genes underexpressed at 30° were also enriched for the functional categories secondary metabolic process, oxidoreductase activity, extracellular region, and toxin metabolic process, but not for the cell wall and carbohydrate metabolic process categories (Figure 2A and File S3).

In total, 274 genes were overexpressed in both Δ*veA* and Δ*laeA* at 37° (17% of all genes overexpressed in Δ*veA* and 43% of all genes overexpressed in Δ*laeA*), while 104 genes were overexpressed in both Δ*veA* and Δ*laeA* at 30° (10% of all genes overexpressed in Δ*veA* and 23% of all genes overexpressed in Δ*laeA*) (Figure 2, C and D). Enrichment of functional categories in genes that were overexpressed in Δ*veA* and Δ*laeA* was strikingly different at 30 and 37°. Although the categories RNA metabolic process, DNA binding, helicase activity, nucleus, DNA-dependent transcription, regulation of biological process, protein kinase activity, chromosome, hydrolase activity, and DNA metabolic process were significantly enriched in genes overexpressed in both Δ*veA* and Δ*laeA* at 37° (Figure 2A and File S3), no functional categories were significantly enriched at 30°. Because fewer genes were overexpressed than were underexpressed in both Δ*veA* and Δ*laeA* at either temperature, and many more genes were overexpressed in VeA’s absence than in LaeA’s absence, these results suggest that LaeA may primarily function as a positive regulator of gene expression.

### Many SM gene clusters are regulated by both VeA and LaeA at 37°, but only by LaeA at 30°

**Figure 3:**
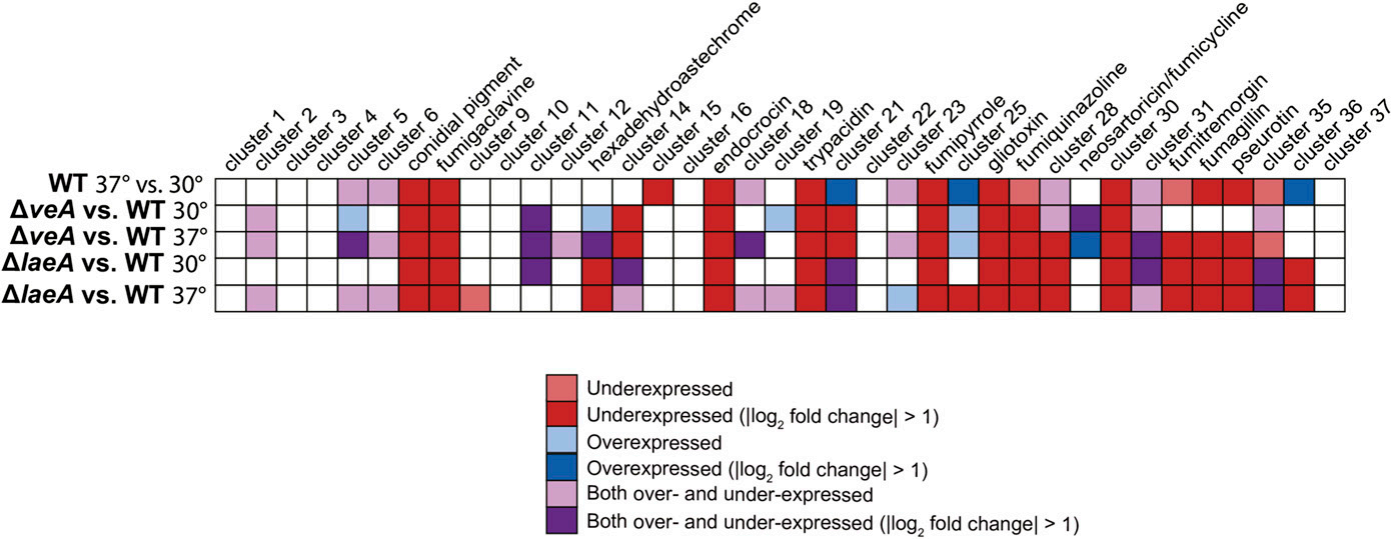
Differential expression of SM gene clusters in all conditions. Dark red boxes indicate half or more genes are underexpressed, dark blue boxes indicate half or more genes are overexpressed, and dark purple boxes indicate that half or more genes are a combination of overexpressed and underexpressed genes. Light-colored boxes indicate that half or more genes in that gene cluster meet the statistical significance cutoff for differential expression but have less than a twofold change in expression.

**Figure 4:**
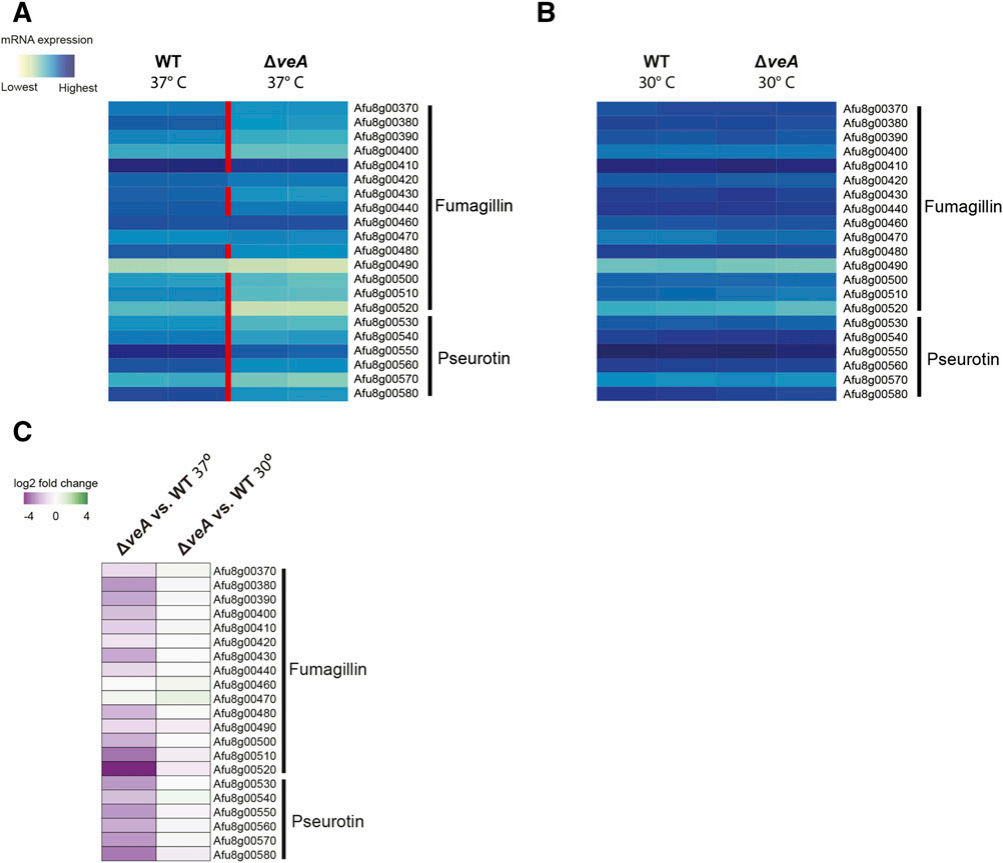
Expression (A and B) and differential expression (C) of the fumagillin and pseurotin clusters in wild-type and Δ*veA* at 37° and 30°. (A and B) Expression is shown as the regularized log transformation of the number of RNA-seq reads aligning to that gene. Genes that are under-expressed in Δ*veA* are separated by a red line. (C) log_2_ fold change of all genes in the fumagillin and pseurotin gene clusters between Δ*veA* vs. WT at 37° and 30°.

We expect that SM clusters regulated by the Velvet complex, comprised of the VelB, VeA, and LaeA proteins (Bayram *et al.* 2008), will require both VeA and LaeA for WT levels of expression. SM gene clusters not controlled by this protein complex, however, may not show differential gene expression in Δ*veA* or Δ*laeA* strains, or may be differentially expressed in only one strain. At 37°, 12 SM gene clusters were underexpressed in both Δ*veA* and Δ*laeA*, suggesting that they may be regulated by the Velvet protein complex; these clusters include 1,8-dihydroxynaphthalene (DHN) melanin pigment, fumigaclavine, endocrocin, trypacidin, fumipyrrole, gliotoxin, fumiquinazoline, cluster 28, cluster 30, fumitremorgin, fumagillin, and pseurotin (Figure 3). Interestingly, six of these SM gene clusters were either normally expressed in Δ*veA* at 30° or had much less of a change from WT expression, suggesting that VeA’s regulatory role may be temperature-dependent. These clusters include DHN melanin pigment, fumiquinazoline, cluster 28, fumitremorgin, fumagillin, and pseurotin (Figure 3, Figure 4, Figure S1, and File S1). Furthermore, clusters that expressed more highly at 37° than at 30° in WT *A. fumigatus* were also often underexpressed in Δ*veA* and Δ*laeA* strains; of the 12 clusters underexpressed in both Δ*veA* and Δ*laeA* strains at 37° (Figure 3), 11 were expressed at higher levels in WT at 30° than at 37°. The exception was cluster 28, which was underexpressed in both Δ*veA* and Δ*laeA* strains at 37° but was expressed at similar levels in WT at 30 and 37°.

Several clusters were differentially expressed in either Δ*laeA* or Δ*veA*, but not in both. The hexadehydroastechrome cluster, while underexpressed in Δ*laeA* at both 30 and 37°, was overexpressed in Δ*veA* at 30° and showed mixed expression in Δ*veA* at 37° (Figure 3). Two gene clusters, cluster 14 and cluster 21, were underexpressed in Δ*veA* at both temperatures but showed mixed expression in Δ*laeA*. Finally, the neosartoricin/fumicycline cluster, which was very lowly expressed in WT at both 30 and 37°, contained some up-regulated genes in Δ*veA* at both temperatures, but showed no change in expression in Δ*laeA* strains. The expression patterns of these gene clusters indicate that, although VeA and LaeA play roles in their regulation, these proteins may in some cases be acting independently of each other.

## Discussion

Production of SMs in *A. fumigatus* and other filamentous fungi is triggered by diverse environmental cues, such as temperature, pH, and nutrient sources, and several master SM regulators that respond to these cues have been identified. However, the extent to which master SM regulators can respond to multiple environmental cues to regulate SM production is not known. Considered together, our findings that temperature regulates global SM production in *A. fumigatus* and that the light-responsive master SM regulator VeA is also responsive to changes in temperature, provide support for the hypothesis that regulation of SM production occurs in response to multiple environmental cues.

Growth at 37° Compared with 30° had a marked impact on gene expression in *A. fumigatus* WT, significantly changing the expression levels of ∼10% of its genes. Importantly, genes involved in secondary metabolism were disproportionately affected (Figure 1A); 13 of the total 37 SM gene clusters were expressed at higher levels at 30° than at 37°, while three clusters were expressed at lower levels at 30° (Figure 3A). These results are in accordance with studies in *A. flavus* that find a global pattern of higher SM cluster expression at 30° than at 37°, the optimal temperature for growth in both fungi (Yu *et al.* 2011). Additional support for our findings that temperature plays a significant role in SM gene expression was provided by qRT-PCR assays of two SM genes, gliP and psoA, in temperature-shift experiments (Figure S3). Specifically, a temperature shift from 37 to 30° increased the expression of both genes, supporting our conclusion that temperature modulates SM gene expression.

To elucidate the effects of temperature on SM regulation, we exposed deletion strains of genes encoding two key members of the Velvet protein complex, *veA* and *laeA*, to different temperature conditions. At 37°, the optimal temperature for *A. fumigatus* growth, we find that VeA and LaeA are both involved in regulating genes in many SM gene clusters. While the lists of which genes are parts of the known SM gene clusters are not identical to the lists used in previous analyses of LaeA’s regulatory role of controlling secondary metabolism, our RNA-seq results generally agree with previously published microarray data (Perrin *et al.* 2007). One notable difference from previous reports is our finding that a putative terpene-producing cluster on chromosome 5 (Afu5g00100–00135) is under LaeA regulation. Further, our findings that VeA transcriptionally regulates many gene clusters agrees with chemical data that show that VeA is required for the synthesis of fumagillin, fumitremorgin, and fumigaclavine at 37° (Dhingra *et al.* 2013).

The sets of genes that are increased in VeA and LaeA’s absence do not show broad overlap in their functions (Figure 1A), suggesting that VeA and LaeA’s regulatory roles are distinct from each other. This inference is further supported by the observation of six SM gene clusters that are differentially regulated by VeA but not by LaeA at different temperatures. The Velvet protein complex formed by LaeA, VeA, and VelB has been implicated as a regulator of secondary metabolism in many fungi (Bayram *et al.* 2008; Calvo 2008; Wiemann *et al.* 2010; Bayram and Braus 2012; Chettri *et al.* 2012); these data provide additional evidence that the LaeA and VeA have functionally distinct roles in regulating SM clusters (Bayram and Braus 2012; Lin *et al.* 2013).

Our finding that VeA’s regulation of SM gene clusters is temperature-dependent raises the hypothesis that, in addition to its critical role in controlling dark-responsive secondary metabolism by localizing in the nucleus under dark conditions and, to a lesser degree, under light conditions (Bayram *et al.* 2008), VeA may also be involved in controlling the response to temperature. Interestingly, previous work in *A. nidulans* has shown that glucose concentration influences both VeA’s subcellular localization and sterigmatocystin production, altering the effect of light on the biosynthesis of this mycotoxin (Atoui *et al.* 2010); thus, light and temperature might just be two of the many environmental cues to which VeA responds.

How might VeA, a single protein, mediate such a diversity of regulatory controls on multiple SM gene clusters in response to several different environmental cues? One possibility is that VeA’s regulatory diversity is mediated through the protein’s multiple interaction partners. VeA forms a heterodimer with another Velvet family protein, VelB, and both proteins are necessary for sexual fruiting body formation in *A. nidulans*. Many of VeA’s interacting partners impact its subcellular localization. For example, in *A. nidulans* VeA interacts with the methyltransferases LlmF and the VipC–VapB heterodimer, which respectively increase and repress VeA’s nuclear import (Palmer *et al.* 2013; Sarikaya-Bayram *et al.* 2014). VeA is also known to interact directly with the red light sensing protein FphA and therefore indirectly with the blue light sensing White Collar homologs LreA and LreB, which may modulate VeA’s light responsive capabilities and subcellular location, as well as potentially playing a role in glucose response (Purschwitz *et al.* 2008, 2009; Atoui *et al.* 2010; Sarikaya-Bayram *et al.* 2015). Another possible mechanism explaining VeA’s multifaceted role is offered by recent experiments in *A. nidulans* showing that phosphorylation of different combinations of residues of VeA generates distinct phenotypes, including changes in sterigmatocystin production (Rauscher *et al.* 2015).

Irrespective of what the precise molecular mechanism(s) contribute to VeA’s diverse array of regulatory controls, the emerging picture from recent studies, including this one, is that VeA is responding to multiple environmental signals, including light (Bayram *et al.* 2008), glucose (Atoui *et al.* 2010), nitrogen source (López-Berges *et al.* 2014), and temperature (this study), allowing filamentous fungi to modulate cellular processes such as secondary metabolism in response to changing environments.

## Supplementary Material

Supplemental Material
