# Supplementary material for: Regulation of Secondary Metabolism by the Velvet Complex Is Temperature-Responsive in Aspergillus
Source: G3 (Bethesda). 2016 Sep 30;6(12):4023–33. doi: 10.1534/g3.116.033084 (PMC5144971; doi:10.1534/g3.116.033084)
Supplement: Supplemental Material [file supp_6_12_4023__index.html]

Regulation of Secondary Metabolism by the Velvet Complex Is Temperature-Responsive in Aspergillus — Supplemental Material 

# Regulation of Secondary Metabolism by the Velvet Complex Is Temperature-Responsive in *Aspergillus*

## Supplemental Material for Lind, *et al*, 2016

**Files in this Data Supplement:**

- Figure S1 - Expression and differential expression of gene clusters showing a higher change in gene expression in Δ*veA* 37° than at 30°. (.pdf, 54 KB)
- Figure S2 - Expression analysis of veA and laeA in *A. fumigatus* wild type and respective mutants at 30° and 37° by qRT-PCR. (.pdf, 86 KB)
- Figure S3 - Expression analysis of (A) gliP and (B) psoA in during temperature shifts. (.pdf, 25 KB)
- Table S1 - qRT-PCR primers used for temperature shift experiments. (.pdf, 168 KB)
- File S1 - Differential expression of all temperature comparisons, including wildtype 37°C vs 30°C, ΔveA vs WT 30°C, ΔveA vs WT 37°C, ΔlaeA vs WT 30°C, and ΔlaeA vs WT 37°C. (.xlsx, 2,195 KB)
- File S2 - Significantly enriched GO categories for all differential expression comparisons. (.xlsx, 68 KB)
- File S3 - Functional enrichment of genes differentially expressed in both ΔveA and ΔlaeA at different temperatures. (.xlsx, 111 KB)
