# Supplementary material for: Regulation of Secondary Metabolism by the Velvet Complex Is Temperature-Responsive in Aspergillus
Source: G3 (Bethesda). 2016 Sep 30;6(12):4023–33. doi: 10.1534/g3.116.033084 (PMC5144971; doi:10.1534/g3.116.033084)
Supplement: Supplemental Material [file supp_g3.116.033084_FigureS1.pdf]

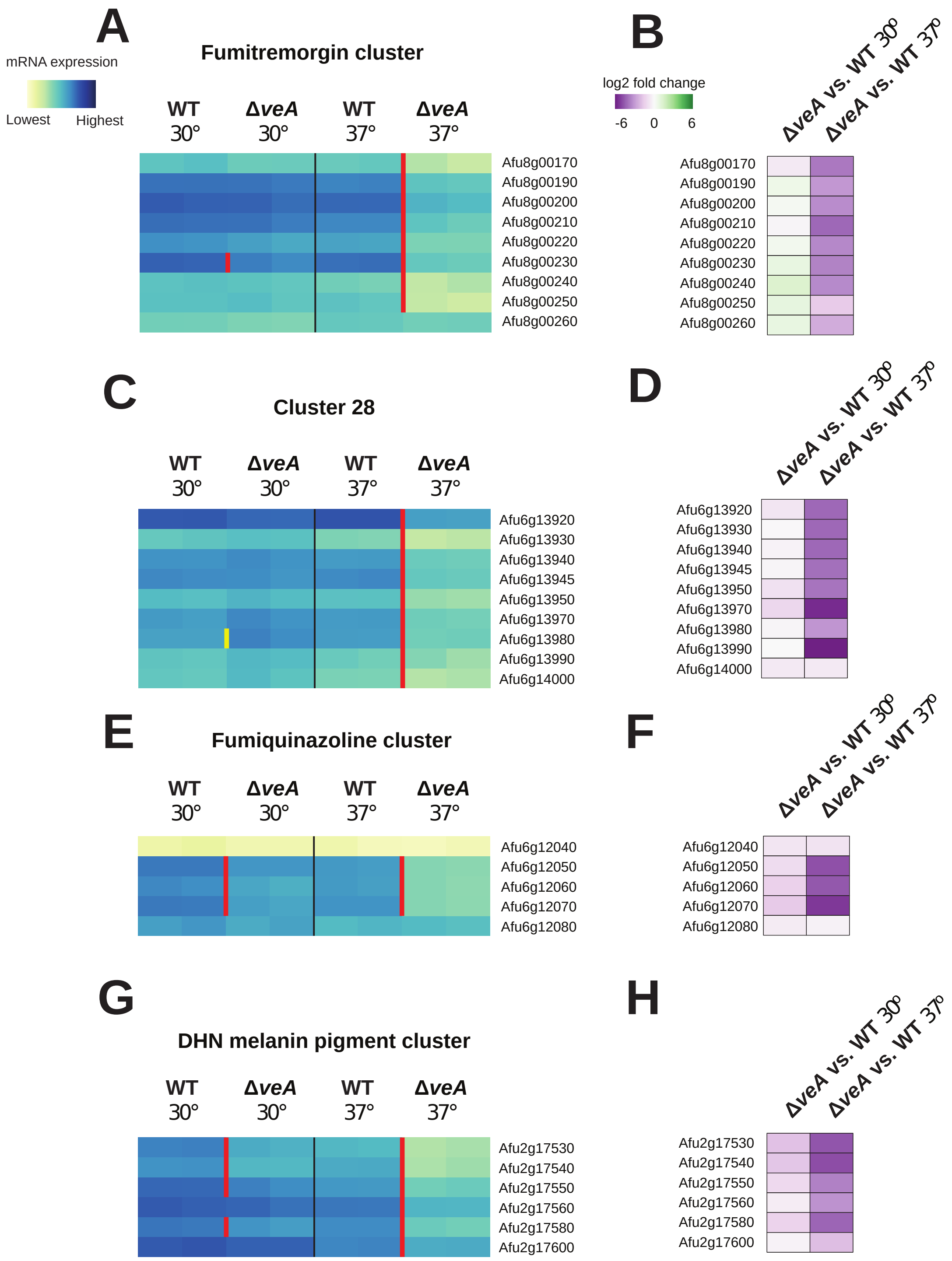

Figure S1 Expression and differential expression of gene clusters showing a higher change in gene expression in Δ*veA* 37º than at 30º. (A,B) Fumitremorgin cluster, (C,D) cluster 28, (E,F), fumiquinazoline cluster, and (G,H) DHN melanin pigment clusters in wild-type and ΔveA at 37º and 30º. Expression (A,C,E,G) is represented as the regularized log transformation of the number of RNA-seq reads aligning to that gene as implementedin DESeq2. Genes that are differentially expressed between conditions are separated by a red line if under-expressed in Δ*veA* or yellow line if over-expressed in Δ*veA*. Differential expression between ΔveA and wild-type is also shown as $\log_2$ fold change (B,D,F,H).
