# Supplementary material for: Regulation of Secondary Metabolism by the Velvet Complex Is Temperature-Responsive in Aspergillus
Source: G3 (Bethesda). 2016 Sep 30;6(12):4023–33. doi: 10.1534/g3.116.033084 (PMC5144971; doi:10.1534/g3.116.033084)
Supplement: Supplemental Material [file supp_g3.116.033084_FigureS2.pdf]

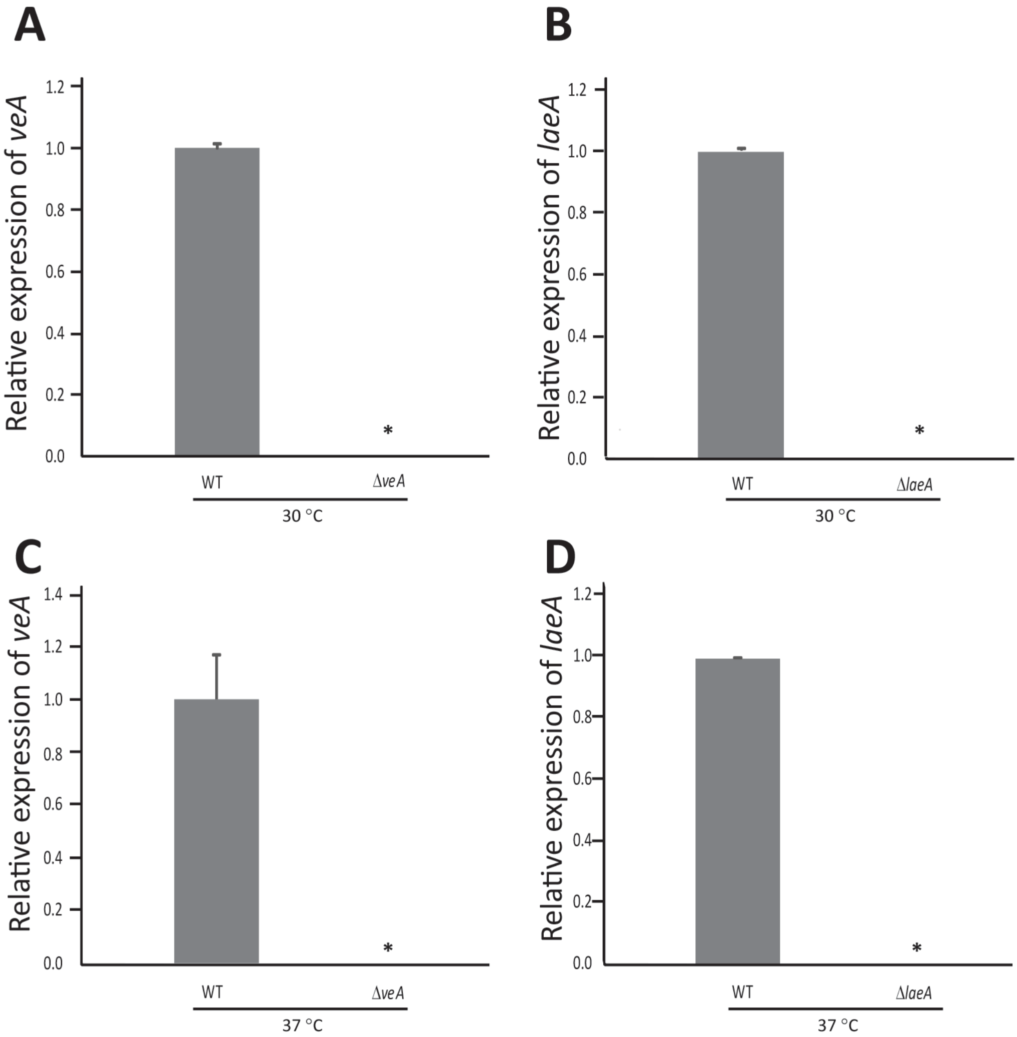

Figure S2. Expression analysis of veA and laeA in A. fumigatus wild type and respective mutants at 30° and 37° by qRT-PCR. Strains were grown on Czapek Dox medium. Expression was normalized to the wild-type. Bars represent standard error. Asterisks indicate no detection.
