# Supplementary material for: Regulation of Secondary Metabolism by the Velvet Complex Is Temperature-Responsive in Aspergillus
Source: G3 (Bethesda). 2016 Sep 30;6(12):4023–33. doi: 10.1534/g3.116.033084 (PMC5144971; doi:10.1534/g3.116.033084)
Supplement: Supplemental Material [file supp_g3.116.033084_FigureS3.pdf]

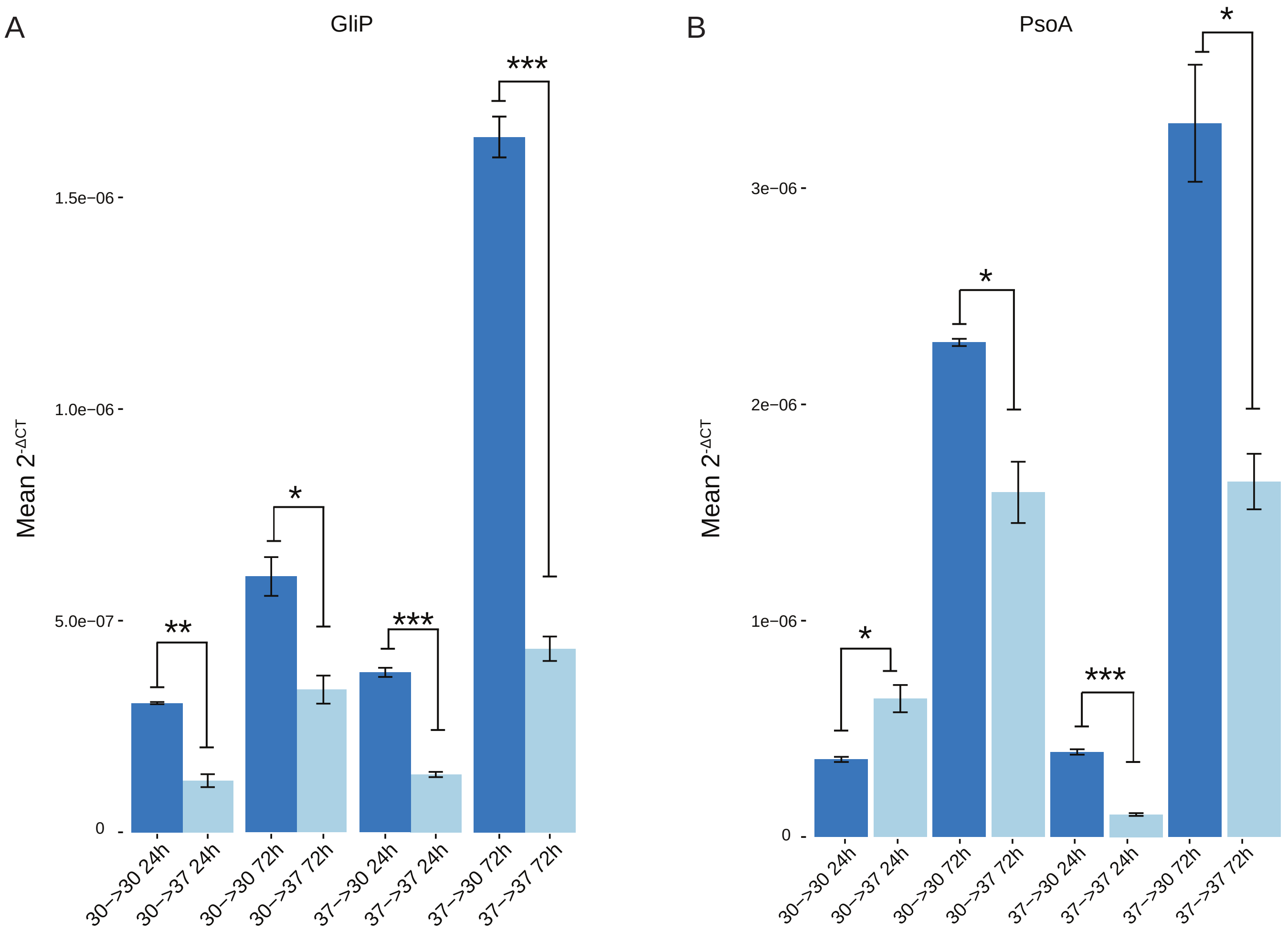

Figure S3 Expression analysis of (A) gliP and (B) psoA in during temperature shifts. Wild-type A. fumigatus was cultured at 30º and 37º for 24 hours, then shifted to either 30º or 37º for 24 or 72 hours as indicated. Three biological replicates were performed for each sample. Error bars represent standard error. Statistical comparisons between conditions were performed using Welch's t-test. Asterisks indicate statistical significance; one asterisk indicates $p < 0.05$, two asterisks indicates $p < 0.01$, and three asterisks indicate $p < 0.001$
