# Supplementary material for: Regulation of Secondary Metabolism by the Velvet Complex Is Temperature-Responsive in Aspergillus
Source: G3 (Bethesda). 2016 Sep 30;6(12):4023–33. doi: 10.1534/g3.116.033084 (PMC5144971; doi:10.1534/g3.116.033084)
Supplement: Supplemental Material [file supp_g3.116.033084_TableS1.pdf]

Table S1. qRT-PCR primers used for temperature shift experiments.

Primer	Sequence
**18sAfum_F**	TAGTCGGGGGCGTCAGTATTCAGC
**18sAfum_R**	GTAAGGTGCCGAGCGGGTCATCAT
**Afum_gliP_F**	AGTTACACCGACTCGCATCCAGC
**Afum_gliP_R**	CTGGGGCAGACCATGCGTAG
**Afum_psoA_F**	CTCTGGCGGCGAGATTGGTT
**Afum_psoA_R**	CCGCCCTTCTTTCCATCCTTCC
